# Single-Shot Smartphone-Based Quantitative Phase Imaging Using a Distorted Grating

**DOI:** 10.1371/journal.pone.0159596

**Published:** 2016-07-21

**Authors:** Zhenyu Yang, Qiwen Zhan

**Affiliations:** Department of Electrical & Computer Engineering and Electro-Optics Program, University of Dayton, Dayton, Ohio, United States of America; Tufts University, UNITED STATES

## Abstract

Blood testing has been used as an essential tool to diagnose diseases for decades. Recently, there has been a rapid developing trend in using Quantitative Phase Imaging (QPI) methods for blood cell screening. Compared to traditional blood testing techniques, QPI has the advantage of avoiding dyeing or staining the specimen, which may cause damage to the cells. However, most existing systems are bulky and costly, requiring experienced personnel to operate. This work demonstrates the integration of one QPI method onto a smartphone platform and the application of imaging red blood cells. The adopted QPI method is based on solving the Intensity Transport Equation (ITE) from two de-focused pupil images taken in one shot by the smartphone camera. The device demonstrates a system resolution of about 1 μm, and is ready to be used for 3D morphological study of red blood cells.

## Introduction

Blood sample test and analysis plays an extremely important role in diagnosis of many diseases. However, conventional blood testing technologies usually rely on large-scale, heavy-duty and expensive optical instruments, as well as trained physicians to operate them. Additionally, preparing blood samples for these tests often involves staining or dyeing the blood smears, which makes the procedure more complicated. In many developing countries and rural areas, it is very common that the absence of both the equipment and well-trained expertise limits the availability of blood tests. Even in more developed areas, blood tests are also prevented from the general public’s daily usage for healthcare purposes. As such, more compact, easy-to-use and cost-effective devices are highly desirable. These devices could be a solution to the emerging needs of blood tests in less developed areas of the world, or as point-of-care systems to perform rapid tests for patients who live in more developed regions.

In the past decade, QPI has been considered a very promising solution to image transparent biological specimens, such as blood cells [1–15], which are essentially phase objects. QPI is label-free as it is aimed to capture the phase information of the object, while the traditional techniques require labeling of the bio-specimen that usually kills the cells. In addition, unlike some of the other techniques, e.g. fluorescence imaging, that use ultraviolet light, which also cause damage to the live cells, most QPI techniques use visible illuminations. More importantly, QPI detected phase maps can be further processed into cell biomechanical data, such as 3-D distributions of refractive index and the membrane fluctuations in red blood cells [14]. These data contain large amounts of information, which is not correlated in the traditional microscopic sensing techniques. Using different interferometric QPI methods (including FPM, HPM and DPM) to quantitative imaging red blood cell dynamics has been demonstrated with decent sensitivity [15]. Also, various compact and portable QPI systems have been reported recently, including the τ interferometer [16], self-referencing DHM [17] and a common path lateral phase shifting interferometer unit attached to an inverted microscope [18], demonstrating encouraging imaging capability and sensitivity.

In the meantime, another recent rising trend is developing optical imaging and sensing devices for medical purposes based on smartphone platforms [19–21]. These devices focus on implementing the functions of traditional optical instruments on smartphones. Through taking advantages of the already existing smartphone built-in sensors and using additional miniature optics, the size and complexity of the optical imaging and sensing systems can be significantly reduced. Towards this end, different “smartphone microscopy” setups [22–27] as well as implementations of more advanced “smartphone fluorescence microscopies” [28–30] have been reported by several groups. However, most of the mentioned devices only provide the 2D information, yet still did not dispose of the need of dyeing the samples. There are also label-free smartphone-based microscopic setups, such as the smartphone-based rapid blood analyzer designed for counting the density of red blood cells in whole blood samples [31] and the smartphone-based lens-free microscopy based on holographical phase reconstruction [32]. The successful implementation of optical techniques with smartphones blazed a new way of developing compact and portable sensing and diagnostic tools.

This work reports the integration of wavefront curvature sensing, one of the QPI methods, onto a smartphone platform, and demonstrates its successful application in red blood cells imaging. From the computationally reconstructed wavefront profile, morphological information of the red blood cells can be acquired with a single-shot measurement.

## Materials and Methods

### 2.1 Wavefront Curvature Sensing

Wavefront Curvature Sensing (WCS) is an efficient QPI technique with decent sensitivity, requiring only a few basic optical elements [33]. As illustrated later in this section, using a specially designed micro-grating will further reduce the complexity of the conventional WCS system. The physical basis of the WCS technique is that wavefront distortion will cause local intensity to change along the propagating direction. Typically, a concave wavefront, i.e. converging beam, will induce an intensity increase along the propagation direction; while a convex wavefront, i.e. diverging beam, will lead to an intensity decrease along the propagation direction. Mathematically, this property can be described by the Intensity Transport Equation (ITE) [34]:

$$-k\frac{\partial }{\partial z}I=I\cdot \nabla^{2}W+\nabla I\cdot \nabla W,$$ (1)

where I is the local intensity, k is the wavenumber and W is the wavefront surface in the unit of length. Under the assumption of uniform illumination, the second term on the right side vanishes. Consequently, the Laplacian of the wavefront is directly related to local intensity gradient as:

$$\nabla^{2}W=-\frac{k}{I}\frac{\partial }{\partial z}I.$$ (2)

**Fig 1:**
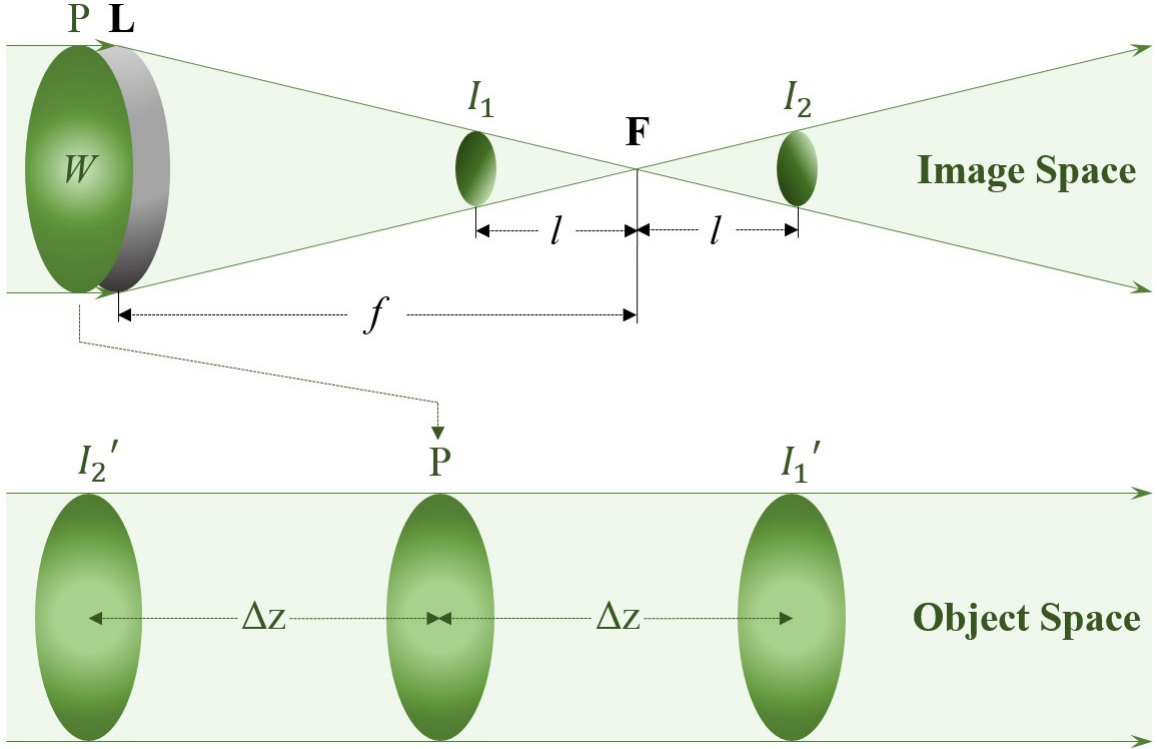
Conjugate planes in the conventional WCS setup.

In the conventional WCS setup [34], the wavefront under test at the pupil is focused onto the focal plane in the image space. Intensity measurements *I*_1_ and *I*_2_ are taken at two planes that are symmetrically located at both sides of the focal plane (Fig 1). From geometric optics, these two measured planes are conjugates of two planes *I*_1_′ and *I*_2_′ in the object space, positioned at both sides of the pupil. Thus the two measurements can be mapped into the object space, for estimating the intensity gradient at the pupil along the propagation direction.

From Newton’s law in geometric optics, Eq (2) can be further evolved to [34]:

$$\nabla^{2}W=\frac{1}{f\left(f-l\right)}\frac{I_{1}-I_{2}}{I_{1}+I_{2}}.$$ (3)

The method to solve Eq (3) is to take Fourier Transforms on both sides of the equation, as the Fourier Transform of wavefront Laplacian on the left side can be written as:

$$FT_{u,v}\{\nabla^{2}W(x,y)\}=-4\pi (u^{2}+v^{2})FT_{u,v}\{W(x,y)\}.$$ (4)

Thereafter, applying an inverse Fourier Transform after combining Eqs (3) and (4) yields the final solution of the wavefront:

$$W=IFT_{x,y}\left\{\frac{FT_{u,v}\left[\frac{l}{f\left(f-l\right)}\frac{I_{1}-I_{2}}{I_{1}+I_{2}}\right]}{-4\pi (u^{2}+v^{2})}\right\}.$$ (5)

### 2.2 Smartphone-based Wavefront Sensor with Micro-gratings

In the conventional WCS setup, the two intensity measurements used as input for the wavefront calculation are located at two different planes. Usually, these images are taken at different times by moving the sensor between the two planes. Such approach requires precise handling/positioning of the system, while the measured images often suffer from the problem of co-registration. More importantly, taking images at different times is not appropriate for some time-variant living bio-samples. To avoid this problem, another option is to use two sensors to take images at the same time with the use of an additional beam splitting optics. However, neither moving the sensing positions nor using bulky beam splitting optics is desirable for developing a portable smartphone-based device.

A solution to the problem is to use a specially designed grating, playing a dual role of both a beam splitter and a de-focuser [35, 36]. As a beam splitter, the grating separates the incoming beam into different diffraction orders and creates multiple images on the same plane, i.e. the camera sensor. On the other hand, the de-focuser role adds additional degrees of defocuses to each diffraction orders. The positive and negative diffraction order images are symmetrically defocused, making them suitable input pairs for the wavefront reconstruction algorithm. Such alternation enables the capture of the desired measurements simultaneously while avoiding the use of traditional bulk beam splitting optics.

A smartphone-based wavefront sensor has been successfully built and verified using the distorted micro-grating approach [37]. The device is able to capture two out-of-focus images (±1^st^ order), along with an in-focus (0^th^ order) image on the smartphone CMOS sensor simultaneously. According to the WCS theory, the two symmetrically out-of-focus images are the two essential parameters for calculating the wavefront surface. In the setup, the 0^th^ order image is used as a reference for focusing the incoming wavefront. The system was also tested with generated known wavefronts, showing a decent sensitivity and accuracy [37].

**Fig 2:**
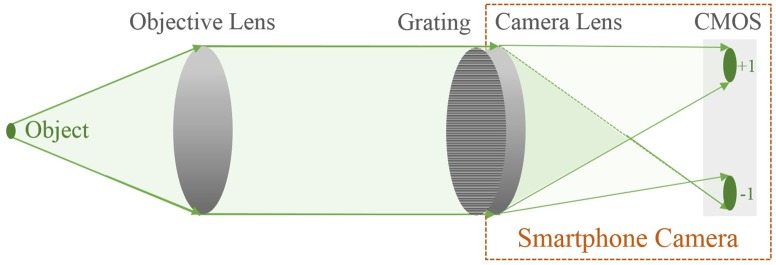
Smartphone-based wavefront sensor with extended system magnification.

**Fig 3:**
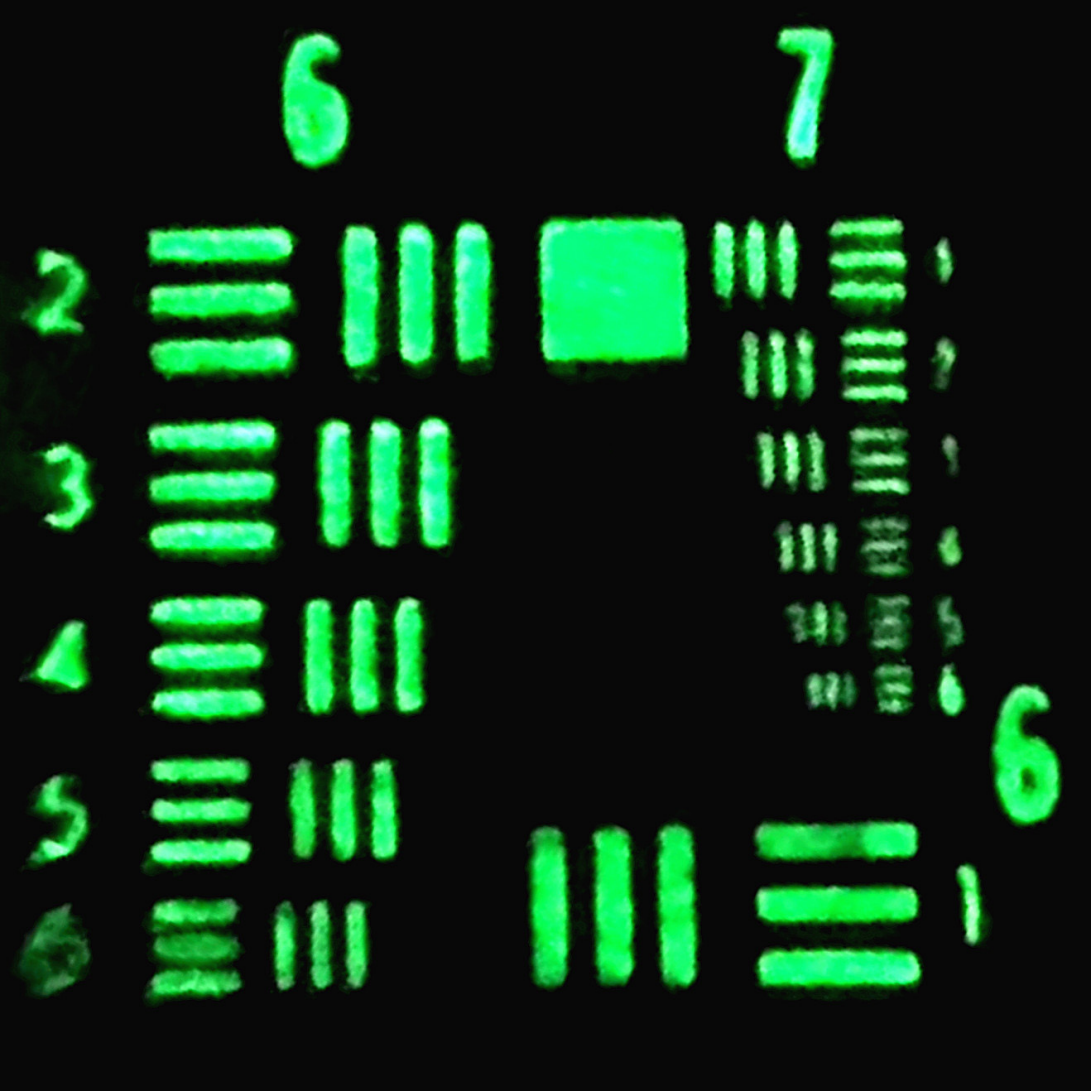
Resulting image of using the smartphone-based system to image the USAF resolution target. The system demonstrates the capability of resolving the 6^th^ element of group 7 on the resolution target, which has a line width around 2 μm.

In order to provide sufficient spatial resolution for red blood cell imaging, the smartphone-based wavefront sensor needs additional magnifying power. To address this problem, an extra lens is introduced to establish a two-lens microscopic system in addition to the smartphone camera lens (Fig 2). In such setup, the external lens is used as the objective lens in a typical microscopic system, while the smartphone camera lens is the eyepiece. The system can be viewed as applying the dual role distorted micro-grating to a smartphone-based microscopy. In our setup, the external lens used is a Thorlabs C330TME-A lens with focal length of 3.1 mm and NA of 0.68, while the focal length of the smartphone camera lens is 4 mm. The system is calibrated and tested to have a resolution around 1 μm (Fig 3), which is sufficient for many bio-specimens. The existing wavefront reconstruction algorithm still applies, whereas the image space measurements need to be mapped to the object space using geometric optics.

### 2.3 Distorted Micro-grating Design and Fabrication

**Fig 4:**
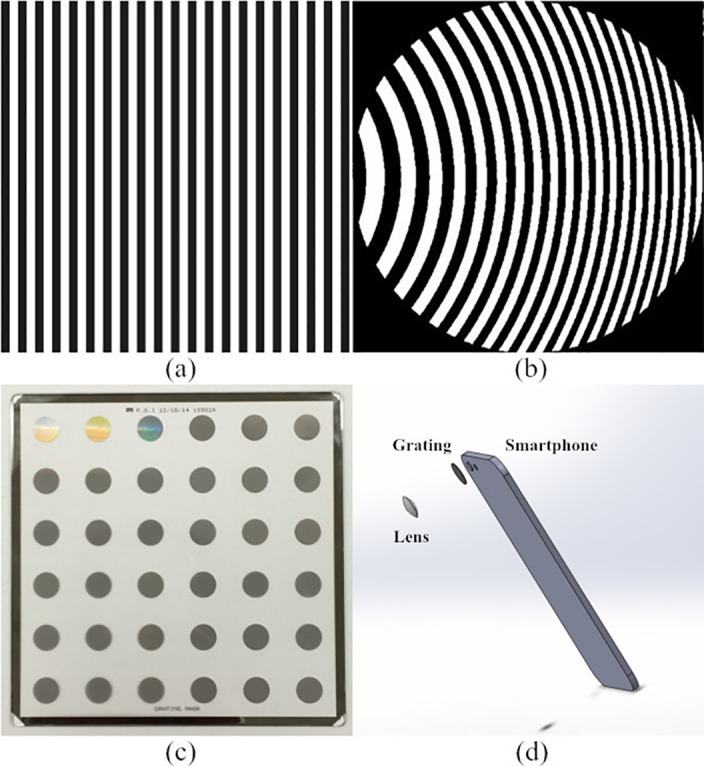
(a) Regular optical grating is consisted of periodical straight lines; (b) Quadratically distorted grating lines in a circular aperture; (c) Fabricated grating masks for smartphone devices, each of the dark circles is a mask; (d) System structure of the prototyping system.

The purpose of using the distorted grating is to separate the incoming wavefront and create multiple images on the same sensor, where each image has a different level of defocus. Regular diffraction gratings with periodical straight grating lines are capable of diffracting lights into different directions. However, to introduce an additional order-related de-focusing, the straight grating lines have to be curved according to specified functions. Such technique of generating desired phase modulation by encoding the optical elements is called binary-encoding. In diffractive optics, focusing and de-focusing effect can be characterized as a quadratic phase shift. Therefore a quadratic distortion can be encoded onto the regular grating lines (Fig 4(B)). It should be noticed that after the quadratic encoding, the local period of the distorted gratings varies spatially across the device aperture. Mathematically, the equation of the grating lines can be expressed as:

$$\frac{x}{d_{0}}+\frac{C(x^{2}+y^{2})}{\lambda R^{2}}=n,$$ (6)

where x and y are the Cartesian coordinates, λ is the wavelength, R is the grating aperture radius, *d*_0_ is the grating period at the aperture center and n refers to the loci of each grating lines. The introduced parameter C controls the degree of curvature of the grating lines [37].

It can be seen from Eq (6), there are two important parameters for designing the grating masks. One of them is the grating period at the mask center *d*_0_, which determines the separating angle between diffraction orders, and therefore controls the distance between the two images on the imaging sensor. The other parameter is the curvature of the quadratic grating lines C, determining degrees of de-focuses associated with each diffraction order. The designed micro-grating masks were fabricated using the Custom Optical Reticle Engraver (CORE) system commercially provided by Photo Science, Inc. based in Torrance, CA. On the sample panel, totally 12 distorted micro-gratings (each has 3 replica) designed with different parameter sets were engraved. The mask paired with a specific smartphone is chosen such that only the ±1^st^ order and the 0^th^ order images fall onto the smartphone CMOS sensor. For the following blood sample test, a micro-grating mask has a grating period of 3.0 μm at the aperture center and the curvature parameter C of 15λ, which creates ±15.6 μm de-focus distances for the ±1^st^ orders away from the 0^th^ order with an Apple iPhone 6.

## Results and Discussion

One typical set of system results includes the raw photo taken by the smartphone, the calculated wavefront and a reconstructed height map. In the current setup, the raw data captured by the smartphone is uploaded onto a server for post processing and calculation. This is accomplished by calling the pre-developed Matlab algorithms on the server by the Matlab mobile app installed on the smartphone. In such a configuration, all the instructions are given from the field smartphone device while the computation is actually running on the much more powerful server, and the computing time to generate one group of results is less than 1 second.

**Fig 5:**
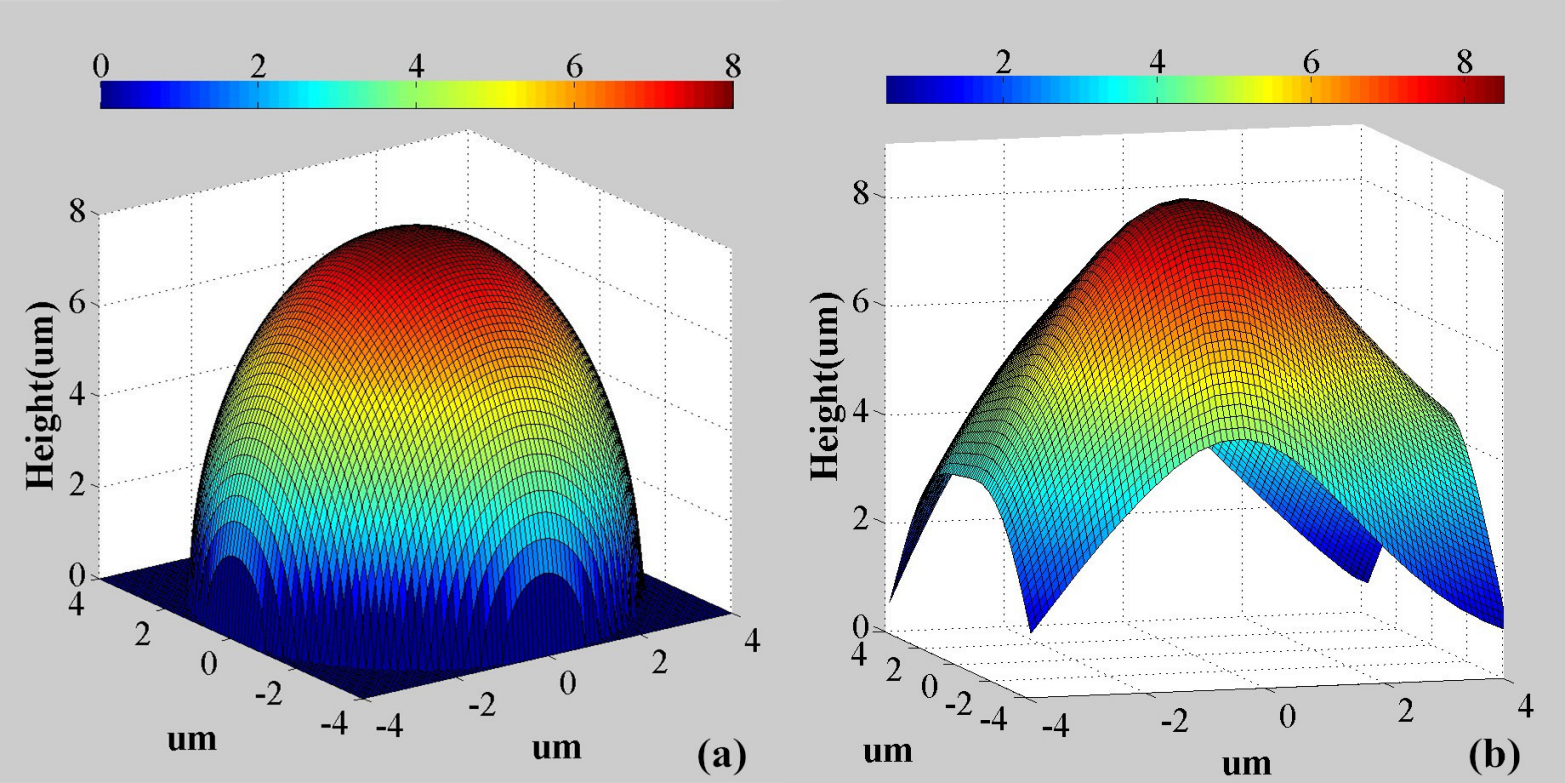
(a) The theoretical thickness map of a 8 μm diameter polymer microsphere; (b) the reconstructed thickness map of the polymer microsphere using the reported system.

The result generated from solving ITE is the phase profile across the scene. There is one more step to convert the calculated phase map into a specimen height map. The relation between sample height and phase can be characterized by

h(x,y)=λϕ(x,y)/2Δn, (7)

where h is the height, ϕ is the phase, *λ* is the illumination wavelength and *Δn* is the refractive index difference between the sample and the surrounding medium. The system was validated by imaging a 8 μm diameter polymer microsphere with refractive index of 1.59. The microspheres are dispersed in distilled water, and the calculated thickness map is provided in Fig 5(B) compared with the theoretical simulation in Fig 5(A). From the results, it can be seen that the measured total height and shape in the central region of the microsphere agrees well with the theoretical predictions. The deviation towards the peripheral region of the microsphere is attributed to extremely high refraction angles for the light illuminating these parts of the sample.

**Fig 6:**
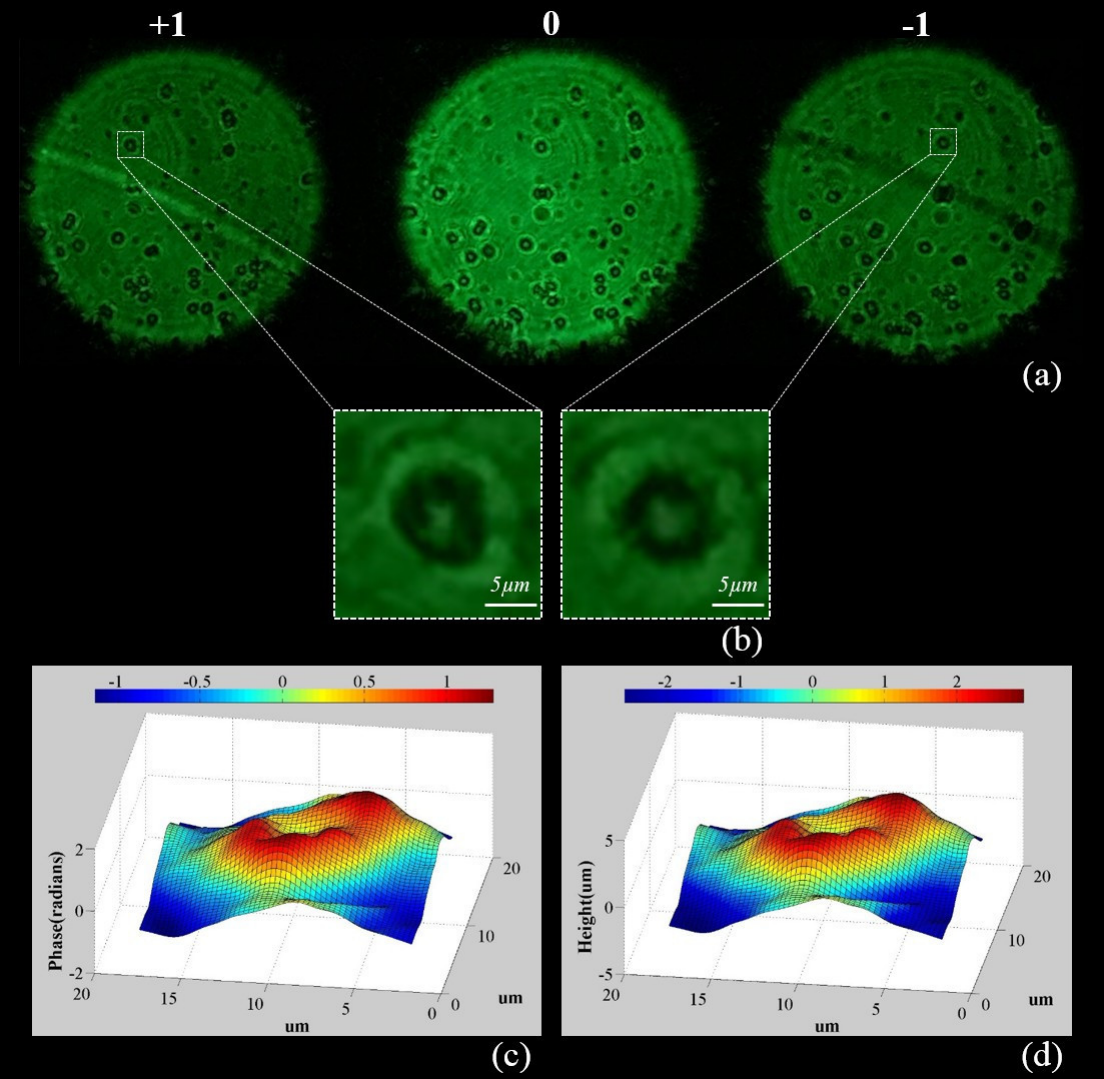
Exemplary results of using the smartphone-based wavefront sensor for red blood cell imaging. (a) A raw photo taken by the smartphone camera, consisting of 3 images of the pupil; (b) Pre-selected and cut images for the wavefront computing algorithm; (c) Calculated phase map; (d) Reconstructed height map.

Fig 6 shows one completed set of data captured for imaging human red blood cells sample using the system. The original photo taken by the smartphone consists of 3 images of the sample, one in-focus in the middle and the other two out-of-focus. As illustrated earlier, the 0^th^ order image is used for focusing the system while the essential data for calculations are the two de-focused images, i.e. the ±1^st^ order images. For the sake of saving computing time and improving the system accuracy, a pre-cutting is processed to select pairs of identical cells from the ±1^st^ order images (see Fig 6(B)). Subsequently, the two pre-cut images are used as the input for the ITE based algorithm to solve the wavefront. The resulted wavefront is then represented as a phase map.

The topography of the red blood cells can be obtained using Eq (7). Practically, the refractive index of red blood cells varies from individuals. Usually, a stricter test requires further examination to determine the red blood cell refractive index for an individual patient [11]. In this work, an average condition is applied only for showing the viability of the technique, where the refractive index of red blood cells is considered to be 1.4 [38]. Compared to the surrounding medium with a refractive index of 1.33, the difference used is 0.07. From a number of tests, the system measured the red blood cells to have thickness around 2–3.2 μm, while the thickness of normal red blood cells at the thickest point has been reported to be 2–2.5 μm [39]. For this particular system, the measuring errors are generally coming from three main aspects, the sensitivity of the smartphone built-in CMOS sensor, the accuracy of the refractive index difference and the wavefront reconstruction algorithm used. Yet, the result is already comparable to most of the QPI blood testing setups introduced earlier that are not smartphone-based.

Although the system shows decent capability and accuracy in the blood cell imaging application, it can be further improved with some system updates. Currently, the reported system is set up in the research lab with a 3D printed frame to hold the fabricated grating panel. However, it can be encapsulated as a portable attachment, where the optical components are integrated and the sample tray can slide in. In such encapsulation, ambient light can be rejected and the grating mask can be mounted more stable and closer to the smartphone camera module, making an excellent point-of-care QPI device and improving the system field-of-view and imaging quality. From the computing perspective, the error of the calculated wavefronts may be further reduced by iteratively applying the Fourier Transforming solution. Moreover, with the video recording capability of smartphone cameras, the reported device has the potential to be extended to a dynamics QPI system.

## Conclusions

ITE based WCS is one of the most efficient QPI methods. The alternation of using a binary-encoded distorted micro-grating in WCS can significantly reduce the system complexity. It has been demonstrated that such technique can be integrated onto a smartphone platform, making an extraordinary field phase imaging device. The magnification of the previously developed smartphone wavefront sensor was improved to the cell level to run blood sample tests. The device demonstrated acceptable capability in imaging red blood cells and reconstructing the thickness of the cells from the calculated phase maps. Compared to the existing systems with similar functionalities, the device is much more compact and cost-effective (Fig 4(D)). Also, operating the system is simplified as taking photos with smartphones, which is much more straightforward than other optical techniques. Moreover, smartphone photos can be uploaded to a distant server via wireless links to be further processed or examined by professionals and physicians. Such device meets needs for developing portable and easy-to-use point-of-care systems, and has the potential to become an integral part of the recent trends of creating global medical big data. With further improvement of the current system, it is expected to have great potentials in many biomedical applications.

## Supporting Information

S1 Fig A raw photo taken by the smartphone camera. (JPG)

S2 Fig Pre-selected and cut images (+1^st^ order). (JPG)

S3 Fig Pre-selected and cut images (-1^st^ order). (JPG)
