# Supplementary figures and images for: Single-Shot Smartphone-Based Quantitative Phase Imaging Using a Distorted Grating
Source: PLoS One. 2016 Jul 21;11(7):e0159596. doi: 10.1371/journal.pone.0159596 (PMC4956142; doi:10.1371/journal.pone.0159596)

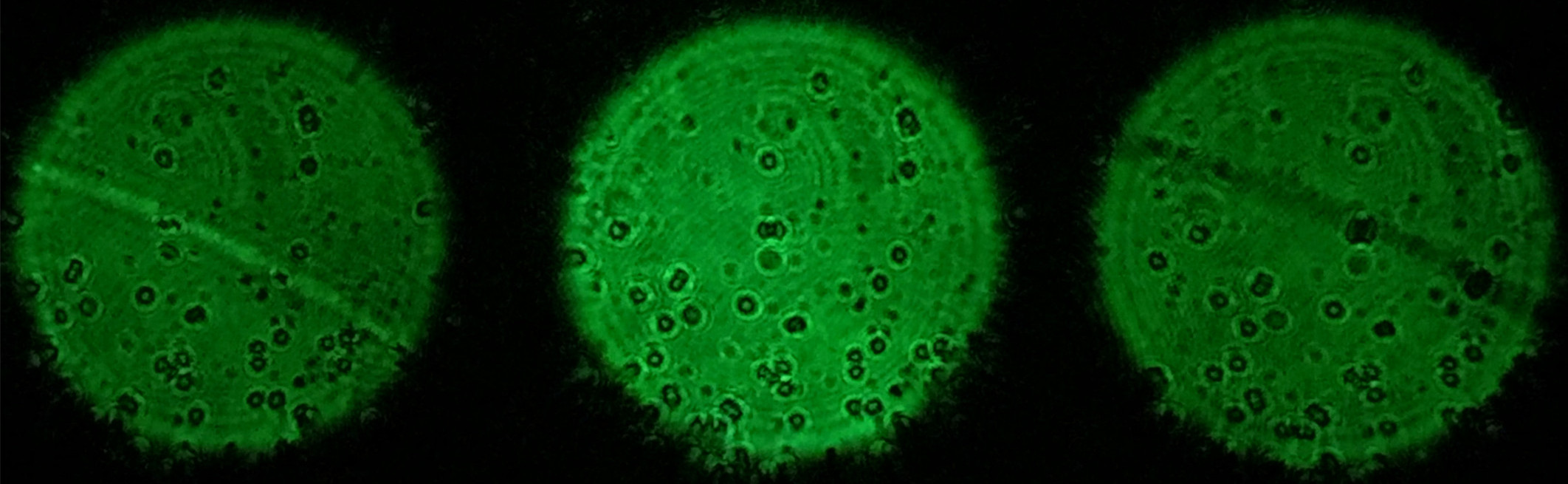

Supplement: S1 Fig — (JPG) [file pone.0159596.s001.jpg]

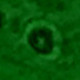

Supplement: S2 Fig — (JPG) [file pone.0159596.s002.jpg]

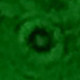

Supplement: S3 Fig — (JPG) [file pone.0159596.s003.jpg]
